# Transcriptome-Based Dissection of Intracranial Aneurysms Unveils an “Immuno-Thermal” Microenvironment and Defines a Pathological Feature-Derived Gene Signature for Risk Estimation

**DOI:** 10.3389/fimmu.2022.878195

**Published:** 2022-05-31

**Authors:** Taoyuan Lu, Zaoqu Liu, Dehua Guo, Chi Ma, Lin Duan, Yanyan He, Rufeng Jia, Chunguang Guo, Zhe Xing, Yiying Liu, Tianxiao Li, Yingkun He

**Affiliations:** ^1^ Department of Cerebrovascular Disease, Zhengzhou University People’s Hospital, Henan Provincial People’s Hospital, Zhengzhou, China; ^2^ Henan Provincial NeuroInterventional Engineering Research Center, Henan International Joint Laboratory of Cerebrovascular Disease, and Henan Engineering Research Center of Cerebrovascular Intervention Innovation, Zhengzhou, China; ^3^ Department of Interventional Radiology, The First Affiliated Hospital of Zhengzhou University, Zhengzhou, China; ^4^ Department of Cerebrovascular Disease, Henan University People’s Hospital, Henan Provincial People’s Hospital, Zhengzhou, China; ^5^ Department of Endovascular Surgery, The First Affiliated Hospital of Zhengzhou University, Zhengzhou, China; ^6^ Department of Neurosurgery, The Fifth Affiliated Hospital of Zhengzhou University, Zhengzhou, China; ^7^ Department of Rehabilitation Medicine, The First Affiliated Hospital of Zhengzhou University, Zhengzhou, China

**Keywords:** intracranial aneurysm, immune microenvironment, machine learning, risk signature, WGCNA, competing endogenous RNA

## Abstract

Immune inflammation plays an essential role in the formation and rupture of intracranial aneurysm (IA). However, the current limited knowledge of alterations in the immune microenvironment of IA has hampered the mastery of pathological mechanisms and technological advances, such as molecular diagnostic and coated stent-based molecular therapy. In this study, seven IA datasets were enrolled from the GEO database to decode the immune microenvironment and relevant biometric alterations. The ssGSEA algorithm was employed for immune infiltration assessment. IAs displayed abundant immune cell infiltration, activated immune-related pathways, and high expression of immune-related genes. Several immunosuppression cells and genes were also coordinately upregulated in IAs. Five immune-related hub genes, including *CXCL10, IL6, IL10, STAT1*, and *VEGFA*, were identified from the protein-protein interaction network and further detected at the protein level. CeRNA networks and latent drugs targeting the hub genes were predicted for targeted therapy reference. Two gene modules recognized *via* WCGNA were functionally associated with contractile smooth muscle loss and extracellular matrix metabolism, respectively. In blood datasets, a pathological feature-derived gene signature (PFDGS) for IA diagnosis and rupture risk prediction was established using machine learning. Patients with high PFDGS scores may possess adverse biological alterations and present with a high risk of morbidity or IA rupture, requiring more vigilance or prompt intervention. Overall, we systematically unveiled an “immuno-thermal” microenvironment characterized by co-enhanced immune activation and immunosuppression in IA, which provides a novel insight into molecular pathology. The PFDGS is a promising signature for optimizing risk surveillance and clinical decision-making in IA patients.

## Introduction

Intracranial aneurysms (IAs) are local pathological dilations at major branches of the cerebral arteries, affecting 3-5% of the adult population worldwide (1). Despite IAs being relatively rare in all stroke subtypes, the sharp pain hit as well as the high risk of early mortality (approximately 18.4%–30%) (2) when progressing to aneurysmal intracranial hemorrhage make them more frightening. As medical imaging, microsurgery, and neurointervention advance by leaps and bounds, unruptured intracranial aneurysms (UIAs) are more frequently detected and prophylactically repaired, greatly ameliorating the patients’ survival (3). However, clinicians are confronted with a dilemma regarding the choice of intervention opportunity, which requires a comprehensive consideration of the risk of rupture and the inherent risks of preventive treatment as well as economic issues. Additionally, endovascular therapy, as the current mainstream radical cure, remained a few uncommon but severe troubles such as (re)hemorrhage and recurrence (4–6). Hence, continued efforts are still oriented toward therapeutic technique improvements and perfection of an individualized risk assessment system, and it is imperative to pursue a more in-depth dissection of the molecular mechanisms.

The formation, progression, and rupture of IAs is an incompletely ascertained pathological process. According to the current understanding, a persuasive point is that immune inflammation plays essential mediating roles in the IA molecular pathology (1, 7, 8). Endothelial injury caused by various risk factors initiates the activation and cascade amplification of inflammation, and then, the local microenvironment is dysregulated, leading to alterations in a variety of biological features encompassing cell dysfunction or loss, extracellular matrix (ECM) destruction and reshaping, and so on. During these processes, accumulated immune cells such as macrophages (9) and immune-related molecules such as interleukin (10) and chemokines (11) are considered to be major mediators. Nevertheless, previous studies only detected a few dysregulated immune cells and immune-related molecules in IAs, lacking a global perspective on the immune microenvironment. With recent strides in genome analysis technology, the immune cellular composition of various human diseases such as atherosclerosis (12), tumors (13), Crohn’s disease (14), and COVID-19 (15) has been described using bioinformatics approaches. Likewise, the immune landscape of IAs, including immune cells and immune-related pathways and molecules, is also expected to be comprehensively revealed. Furthermore, molecular targeted therapy combined with drug-loaded stent technology has made great strides over the past decade, holding new promise for advances in IA treatment. Local immunomodulatory therapies based on various cellular or molecular targets have been demonstrated to efficiently promote neurovascular lesion repair in animal experiments (16–18). In parallel with these technological advances, deeper cognitive demands for microscopic pathological alterations and potential interventional targets of IA have also increased, expecting a systematic exploration of the immune microenvironment and relevant biometric deviations in IAs.

Early diagnosis and aneurysm rupture risk monitoring are vital supports in the clinical decision-making of IAs. Imaging by computed tomographic angiography and digital subtraction angiography is currently the main auxiliary diagnostic method but is not extensively applicable for IA screening because of the high cost, radiation exposure, and invasiveness. In recent years, cumulative studies are also dedicated to exploiting IA rupture relevant predictors [e.g. morphological parameters (19), PHASES score (20), and wall enhancement on MRI (21)] to refine individualized risk assessment systems, but they are mostly based on clinical characteristics or imaging, without regard to molecular pathological features. Various circulating RNAs associated with the lesion molecular pathology are considered promising biomarkers for disease diagnosis or risk estimation (22, 23), while in IAs, relevant exploration is still limited. Thus, further investigation is required to find valuable biomarkers that can undertake IA screening or risk monitoring tasks in a convenient and cost-effective way.

To tackle the aforementioned considerations, the present study attempted to comprehensively explore the immune microenvironment of IAs, decode transcriptome alterations with respect to immunogenomic and biometric traits, as well as develop a pathological feature-derived gene signature (PFDGS) for risk estimation in IAs. Moreover, potential competing endogenous RNA (ceRNA) regulatory networks and therapeutic agents targeting immune-related hub genes were identified to provide targets and drug references for molecular therapy. We also revealed the latent biological significance underlying PFDGS. Overall, our study characterized the local microenvironment and relevant pathological features of IAs and provides an attractive gene signature for auxiliary diagnosis and rupture prediction, which can facilitate mechanism understanding and precision diagnosis and treatment in IAs.

## Materials and Methods

### Publicly Available Data Collection

**Figure 1:**
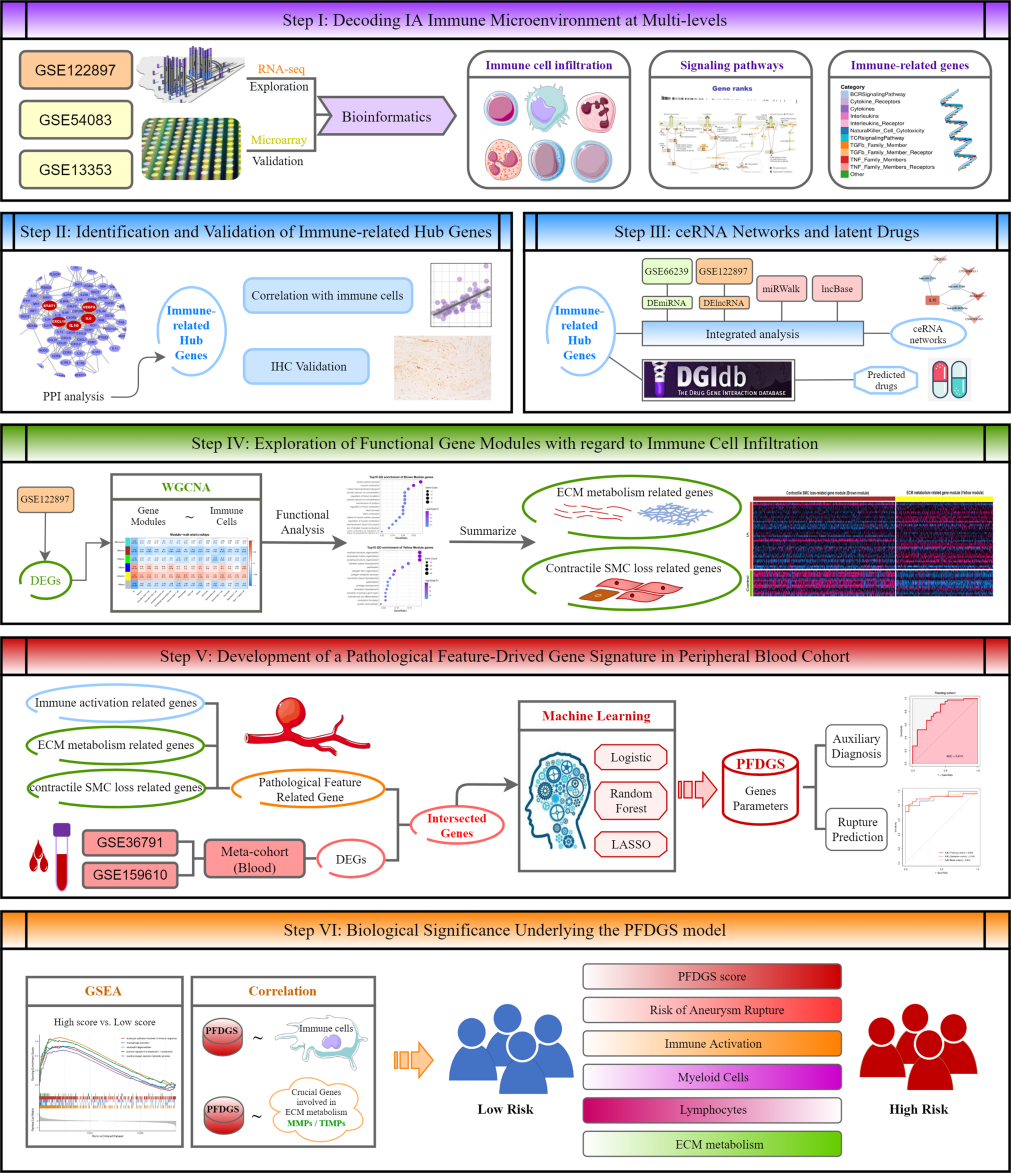
The overall workflow of this study.

The overall workflow of this study is depicted in **Figure 1**. Our study retrospectively recruited seven independent IA cohorts from the Gene Expression Omnibus (GEO, http://www.ncbi.nlm.nih.gov/geo/) database, including GSE122897 (21 ruptured intracranial aneurysm (RIA) tissues, 21 UIA tissues, and 16 control cerebral artery tissues), GSE54083 (13 IA tissues, and 10 control cerebral artery tissues), GSE75436 (15 IA tissues, and 15 control cerebral artery tissues), GSE13353 (11 RIA tissues and 8 UIA tissues), GSE66239 (7 IA tissues and 10 control cerebral artery tissues), GSE36791 (43 blood samples from patients with RIA, and 18 blood samples from control subjects without IA), and GSE159610 (25 blood samples from patients with UIA, and 22 blood samples from control subjects without IA). The detailed baseline information was summarized in **Supplementary Table S1**. Among these cohorts, the GSE122897 dataset was utilized for deciphering immune-relevant alterations in IA local vessels, and GSE54083, GSE75436, and GSE13353 datasets were employed for verification. The precious miRNA array dataset GSE66239 was applied for identifying deregulated microRNA (miRNAs) in IA. A total of 108 blood samples generated by integrating the GSE36791 and GSE159610 datasets were denoted as meta-cohort and utilized to develop and validate a blood-based PFDGS model for IA risk assessment.

### Data Preprocessing and Gene Differential Expression Analysis

For GSE122897, RNA read count data were normalized by variance stable transformation (VST) and further differential expression analyzed using the *DESeq2* R package (24). Genes with an absolute value of log2FoldChange >0.5 and false discovery rate (FDR) <0.05 were considered as differentially expressed genes (DEGs). The microarray data of GSE54083, GSE75436, GSE13353, GSE66239, and GSE36791 datasets were preprocessed *via* quantile normalization and log2 transformation. The RNA-seq raw read counts in GSE159610 were converted to transcripts per kilobase million (TPM) values and further log-2 transformed, which is more similar to those resulting from microarrays and more comparable between samples. The *ComBat* algorithm implemented in the *sva* R package (25) was applied to remove batch effects from non-biological technical biases between GSE36791 and GSE159610 datasets. The principal component analysis (PCA) before and after batch correction was shown in **Supplementary Figure S1**. Ultimately, a total of 14,070 genes were retained in the meta-cohort for subsequent analysis in this study. The GENCODE database (https://www.gencodegenes.org/) was utilized for mRNA and long non-coding RNA (lncRNA) annotations. Gene differential expression analysis of GSE66239 and meta-cohort were performed using *limma* R package (26).

### Estimation of Immune Cell Infiltration

According to the expression level of immune cell-specific marker genes (27), the relative abundance of 28 immune cells in each sample was quantified by single-sample gene set enrichment analysis (ssGSEA) algorithm implemented in the *GSVA* R package (28), which is broadly utilized in immune infiltration-relevant bioinformatics studies (13–15, 29). Please refer to the **Supplementary Table S2** for details of the gene sets marking 28 immune cells.

### Identification of Immune-Related DEGs in IAs

To explore the expression patterns of immune-related molecules in IA, we searched the ImmPort database (https://www.immport.org/) and obtained 1775 immune-related genes (**Supplementary Table 3**) with regard to BCR signaling pathway, TCR signaling pathway, NK cell cytotoxicity, chemokines, cytokines, IFNs, ILs, TGF-b family members, TNF family members, corresponding receptors, and other immune response molecules. Immune-related genes overlapping with DEGs were defined as immune-related DEGs in IAs.

### Enrichment Analysis

In order to ascertain the status of immune-related pathways in IAs, we performed gene set enrichment analysis (GSEA) based on 27 Kyoto Encyclopedia of Genes and Genomes (KEGG) signaling pathway gene sets, which were retrieved from the MSigDB (http://www.gsea-msigdb.org/gsea/msigdb/index.jsp). Detailed pathways and gene lists were depicted in **Supplementary Table S4**. Immune-related DEGs and co-expression module genes were functionally annotated by Gene Ontology (GO) and KEGG enrichment analysis to understand their biological functions. The above procedures were performed using *clusterProfiler* and *fgsea* R packages (30). Permutations were set to 10,000 to obtain normalized enrichment scores (NES) in GSEA. Gene sets with FDR <0.05 were considered significantly enriched. Moreover, the GSEA algorithm was also employed to decipher potential biometric differences between high- and low-risk groups distinguished by PFDGS scores.

### Protein-Protein Interaction Network Analysis of Immune-Related DEGs

The STRING database (https://www.string-db.org/) was applied to generate a PPI network of the immune-related DEGs, which reflects the interaction relationship between the proteins encoded by these genes. Only those interaction pairs with a combined score >0.7 were picked up as remarkable. Hub genes in the PPI network were further identified utilizing the degree algorithm of the cytoHubba plug-in of Cytoscape software.

### Human Specimen Histology and Immunohistochemistry

This study was approved by the Ethical Committee of Zhengzhou University People’s Hospital, China. Five IA tissue specimens and four superficial temporal artery (STA) controls were obtained from patients undergoing IA clipping resection in the Department of Neurosurgery, Zhengzhou University People’s Hospital. All patients were aged >18 years and gave written informed consent.

Paraffin-embedded specimen sections were incubated at 65 °C for 20 min and deparaffinized with xylene and alcohol before staining experiments. Hematoxylin and eosin (HE) stained sections were exploited to ascertain the gross structure of the tissues. For IHC, sections were sequentially incubated with 3% H_2_O_2_, goat serum, primary antibody, biotinylated second antibody, and horseradish enzyme-labeled streptavidin according to the IHC SP kit (ZSGB-BIO: OriGene Technologies, Cat. No. SP-9000) instructions, and then stained by diaminobenzidine (DAB) and hematoxylin. Primary antibodies used in the study were as follows: anti-*CXCL10* (1:500; Proteintech, Cat. No. 10937-1-AP), anti-*IL6* (1:500; Abcam, Cat. No. ab6672), anti-*IL10* (1:500; Proteintech, Cat. No. 20850-1-AP), anti-*STAT1* (1:200; Abcam, Cat. No. ab109320), anti-*VEGFA* (1:100; Abcam, Cat. No. ab52917). IHC results were quantified using the integrated optical density (IOD) method *via* Image-Pro Plus 6.0 software.

### Construction of Potential ceRNA Regulatory Networks and Drug-Gene Interaction Analysis for Hub Genes

The upstream ceRNA regulatory networks of hub genes were constructed through the following pipelines: Firstly, miRNAs targeting five hub genes were predicted *via* miRWalk (http://mirwalk.umm.uni-heidelberg.de/) and filtered with miRWalk Score >0.95. Secondly, differently expressed miRNAs (DEmiRNAs) in IA were identified based on the miRNA array dataset (GSE66239) *via limma* R package with the criteria of abs(log2FoldChange) >0.5 and P-value <0.05. DEmiRNAs overlapping with the predicted miRNAs were considered regulatory miRNAs of hub genes and included in the subsequent analysis. Thirdly, the target lncRNAs of these miRNAs were further predicted according to the lncBase v.2. LncRNAs with abs(log2FoldChange) <0.5 and predicted binding pairs with miTG-score <0.95 were eliminated. Fourthly, the miRNA-mRNA and miRNA-lncRNA binding pairs obtained from the above steps were merged into multiple lncRNA-miRNA-mRNA regulatory axes. Only regulatory axes with significant positive correlations (R >0.3 and P <0.05) between lncRNAs and mRNAs were retained, and then, the ceRNA regulatory networks of the five hub genes were completely constructed.

The potential drugs were predicted *via* DGIdb (https://dgidb.genome.wustl.edu/), an open-source database that converged the known or potential interaction between drugs and genes. Only drug-gene pairs with established interaction types and supported by literature was selected and visualized by Cytoscape software.

### Weighted Gene Co-Expression Network Analysis

The advanced WGCNA method was utilized to identify gene co-expression modules associated with immune cell infiltration. Based on the DEGs between IA and controls, gene co-expression networks were generated using *WGCNA* R package (31). Briefly, genes with similar expression patterns were assigned to a co-expression module based on a weighted correlation adjacency matrix and cluster analysis. An appropriate soft threshold *β* was calculated to meet the criteria for a scale-free network and construct a weighted adjacency matrix. Further, the weighted adjacency matrix was converted into a topological overlap matrix (TOM), and the corresponding dissimilarity was generated (1-TOM). The dynamic tree cutting approach was utilized for module recognition, and modules with less than 0.3 dissimilarities were merged. The relationship between module eigengenes values and immune cell abundance was evaluated *via* Pearson correlation. Modules that were significantly associated with the vast majority of immune cells were considered crucial immune-related modules and were selected for in-depth investigation.

### Integrative Construction of PFDGS in Blood Cohort Using Machine Learning

Peripheral blood DEGs between IAs and controls were identified in the GSE36791 and GSE159610 combined meta-cohort, with *limma-*FDR less than 0.05 as the criteria. Then, peripheral blood DEGs were taken to intersect with dysregulated pathological feature-related genes, comprising upregulated immune-related genes and functional module genes recognized by WGCNA. Genes that presented consistent expression trends in peripheral blood and vascular tissue were incorporated into the model development. Moreover, we transformed gene expression into z-score in the meta-cohort prior to constructing the PFDGS model, which enhanced the comparability between different datasets.

The meta-cohort was randomly divided into a training set and validation set in a 7:3 ratio. The machine learning-based development procedure for the PFDGS model in the training set was as follows: (a) Logistic regression analysis was performed on the pathological feature-related genes, and the genes dramatically associated with IA rupture were selected for subsequent modeling. (b) The random forest algorithm was employed to further screen critical genes, bounded by relative importance greater than 0.5 for variable filtering. (c) Ultimately, the PFDGS signature was constructed using the least absolute shrinkage and selection operator (LASSO) regression based on critical genes recognized from the random forest filter. The PFDGS risk score for each patient was calculated as follows:


PFDGS score = ∑Coef(i)×Expr(i)

where *i* is the key genes obtained from the LASSO regression, *Expr(i)* is the relative expression value (z-score) of *i*, and *Coef(i)* is the regression coefficient of *i*.

The diagnostic and predictive value of critical genes and the PFDGS model were estimated in the training set, validation set, and meta-cohort, respectively.

### Statistical Analysis

All data processing, statistical analysis, and plotting were conducted in R 4.1.0 software. Significance was assessed *via* Student’s *t*-test or Wilcoxon rank-sum test for comparisons of two groups and Kruskal-Wallis test for comparisons of three or more groups. Correlations between two continuous variables were determined using Pearson correlations. Random forest and LASSO regression were implemented *via randomForest* and *glmnet* R packages (32), respectively. Receiver operating characteristic (ROC) curve analysis applied to evaluate the diagnostic or predictive power of variables was performed *via pROC* R package (33). All statistical tests were two-tailed and P <0.05 was considered as statistically significant.

## Results

### The Landscape of Immune Cell Infiltration in IAs

**Figure 2:**
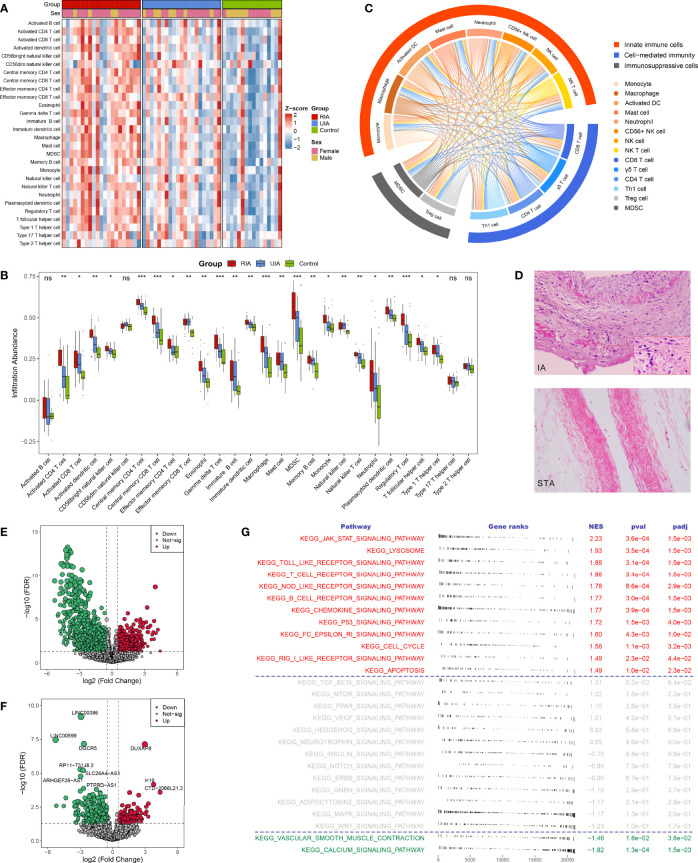
The landscape of immune infiltration and aberrant signaling pathways. **(A, B).** Heatmap **(A)** and comparison boxplot **(B)** of 28 immune cell infiltration between RIA, UIA, and control groups. ns, *P >*0.05; *P < 0.05, **P < 0.01, ***P < 0.001. **(C)** Chord diagram of the correlation of 14 abnormal immune cells. The color line between two cells represented their positive relationship. **(D)** HE staining of IA and STA paraffin sections. Magnification, 100×. **(E, F).** Volcano plots of DEmRNAs **(E)** and DElncRNAs **(F)** between IAs and controls. Red dots indicate up-regulated genes and green dots represent down-regulated genes. **(G)** GSEA analysis of 27 KEGG signaling pathways in IAs and controls. NES >0 indicates pathway activation and NES <0 indicates pathway inhibition. NES, normalized enrichment score.

Using the ssGSEA algorithm, we first measured the relative infiltration abundance of 28 immune cell subpopulations in each sample of the GSE122897 cohort. Overall, most immune cells presented high infiltration levels in IAs, particularly in RIAs (**Figures 2A, B** and **Supplementary Figures S2A, B**), which can be figuratively described as an “immuno-thermal” microenvironment. HE staining revealed the histological features of IAs, mainly comprising internal hemorrhage, structural disorganization, as well as pronounced accumulation of immune cells, which evidenced our inference (**Figure 2D**).

To further ascertain immune cells with stable differential infiltration levels, we additionally assessed immune infiltration in the GSE54083, GSE75436, and GSE13353 datasets (**Supplementary Figures S2C-E**). Compared with normal controls, IAs presented remarkable higher infiltration of 11 effector immune cells, including macrophage, activated dendritic cell (DC), natural killer (NK) cell, NK T cell, CD56+ NK cell, myeloid-derived suppressor cell (MDSC), activated CD4 T cell, activated CD8 T cell, gamma delta (γδ) T cell, regulatory T (Treg) cell, and Type 1 T helper (Th1) cell (**Supplementary Figure S2A, C** and **E**, **Supplementary Table S5**). Moreover, two important inflammatory cells, mast cell, and neutrophil, were also relatively more abundant in IAs, although not stably significant in validation datasets. Further comparison of RIA and UIA detected that the infiltration levels of activated CD4 T cell activated DC, MDSC, γδ T cell, Treg cell, monocyte, and macrophage were elevated in RIAs relative to UIAs (**Supplementary Figures S2B, D** and **Supplementary Table S5**). In addition, no significant differences were observed when contrasting between men and women, suggesting that gender was not an interfering factor (**Supplementary Figure S2F**).

The correlation between immune cells was visualized in the heatmap and chord diagram (**Supplementary Figure S3** and **Figure 2C**). The vast majority of immune cells exhibited a strong positive correlation with each other. Strikingly, among the above 14 vital dysregulated immune cells, two immunosuppressive cells were synergistically elevated with eight innate immune cells and four adaptive immune cells (**Figure 2C**), suggesting a specific microenvironment symptom in IAs: co-existence of immune activation and immunosuppression.

### Aberrant Signaling Pathways and Immune-Related DEGs

In GSE122897, GSEA performed on 27 KEGG signaling pathways showed that immune relevant pathways, such as JAK-STAT signaling pathway, Toll-like receptor signaling pathway, T cell receptor signaling pathway, NOD-like receptor signaling pathway, and chemokine signaling pathway, were remarkably activated in IAs (**Figure 2G**); while smooth muscle contraction relevant pathways encompassing vascular smooth muscle contraction and calcium signaling pathway were pronounced inhibited in IAs. These aberrant signaling pathways characterized the pathological features of immune activation and contractile smooth muscle cell (SMC) dysfunction or loss in IAs and may serve as pathway-based targets candidate for molecular therapy.

**Figure 3:**
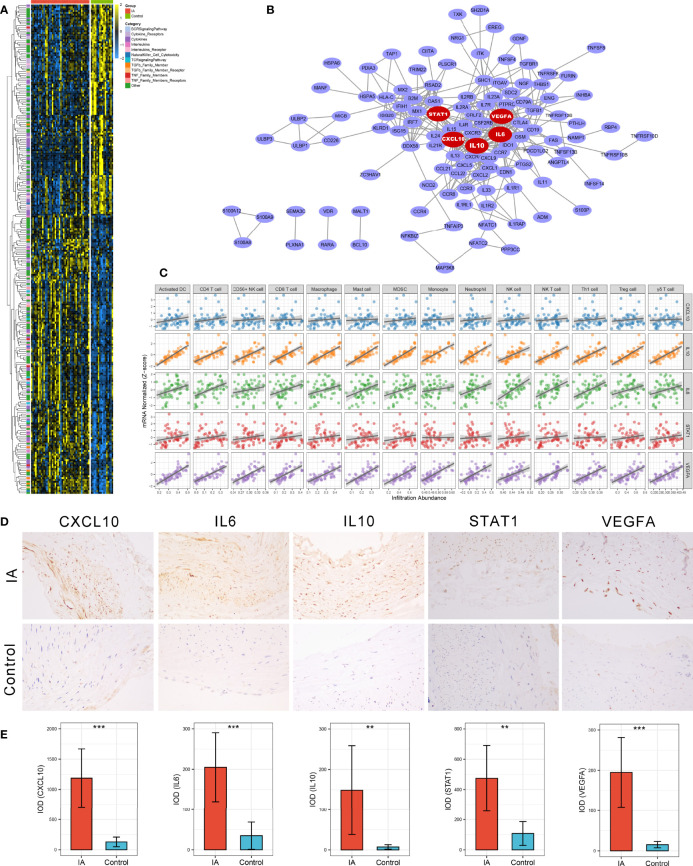
Identification and validation of immune-related hub genes. **(A)** Heatmap of 245 immune-related DEGs. **(B)** Protein-Protein Interaction (PPI) network of upregulated immune-related DEGs. The five red nodes we highlighted in the network were hub genes identified by cytoHubba-degree algorithm. **(C)** Correlation analysis of five hub genes and 14 vital immune cells. **(D)** Representative IHC staining images of CXCL10, IL6, IL10, STAT1, and VEGFA in IAs and STA controls. Magnification, 200×. **(E)** Comparison of protein expression differences of five hub genes according to IHC results. IOD, integrated optical density. **P < 0.01, ***P < 0.001.

A total of 3310 DEGs between IAs and normal controls were identified in GSE122897, including 2746 differentially expressed mRNAs (DEmRNAs, **Figure 2E**), 552 DElncRNAs (**Figure 2F**), and 12 unannotated RNAs. Subsequently, intersecting these DEGs with 1775 immune-related genes collected from the ImmPort, we detected 146 upregulated and 99 downregulated immune-related DEGs in IAs (**Figure 3A**). GO and KEGG enrichment analysis indicated that biological processes with respect to proliferation and activation of various leukocytes, cell chemotaxis and migration, and regulation of cytokine production, as well as immune relevant pathways such as cytokine-cytokine receptor interaction and JAK-STAT signaling pathways, were remarkably enriched in upregulated immune-related DEGs (**Supplementary Figures S4A, C**); whereas the downregulated genes were dramatically associated with the regulation of growth and development (**Supplementary Figures S4B, D**). We thus speculated that those upregulated immune-related DEGs might be major effectors mediating immune activation in IAs and deserved further attention.

### Identification and Validation of Immune-Related Hub Genes

A PPI network reflecting the association of proteins encoded by these upregulated immune-related DEGs was generated *via* the STRING database (**Figure 3B**). Using the cytoHubba plug-in of Cytoscape software, we extracted the Top5 genes ranked by degree method from the PPI network as immune-related hub genes, comprising *IL6, IL10, STAT1, CXCL10*, and *VEGFA*. As well-known, the expression products of these five hub genes are essential immunological molecules involved in immune response regulation. Moreover, correlation analysis indicated that all five hub genes were positively correlated with the 14 vital dysregulated immune cells we highlighted above, implying the immune cell recruitment potential of these hub genes (**Figure 3C**). Notably, anti-inflammatory cytokine *IL10* was pronounced upregulated in IA and strongly correlated with immune infiltration, which suggested a microenvironment feature similar to our cell-based inference that immunosuppression and immune activation coexisted in IAs. To further verify the differential expression of hub genes at the protein level, we performed IHC on paraffin sections from five IA and four STA control samples. Likewise, the protein expression level of *CXCL10* (*P <*0.001), *IL6* (*P <*0.001), *IL10* (*P* =0.008), *STAT1* (*P* =0.002), and *VEGFA* (*P <*0.001) were remarkably higher in IAs (**Figures 3D, E**).

### CeRNA Regulatory Networks and Latent Drugs Targeting Hub Genes

**Figure 4:**
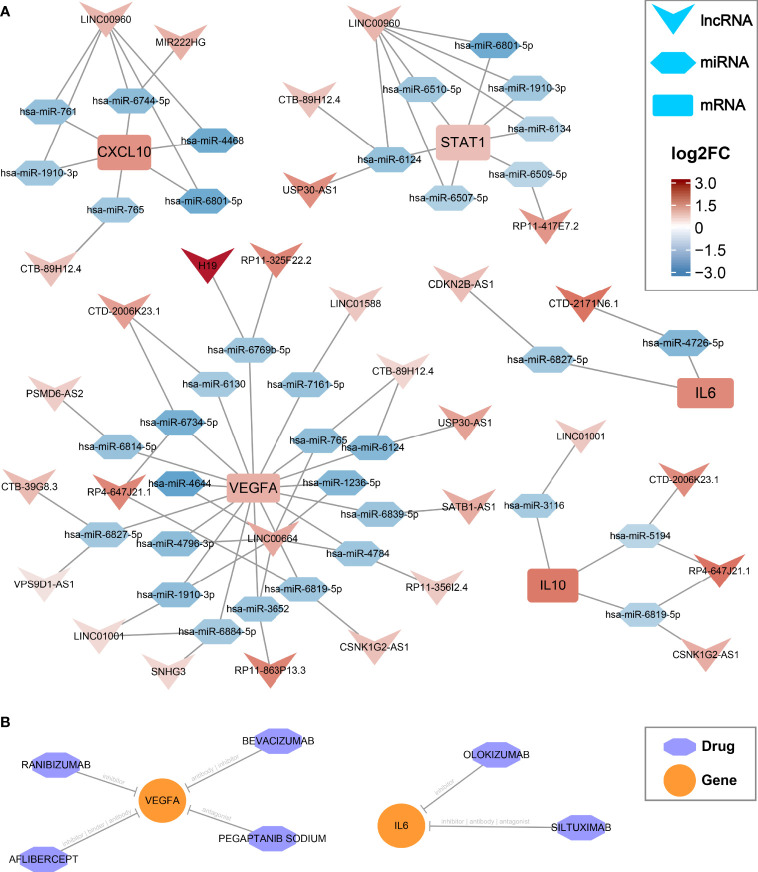
CeRNA regulatory networks and latent drugs targeting the hub genes. **(A)** ceRNA networks of CXCL10, IL6, IL10, STAT1, and VEGFA. Log2FC, log2(foldchange). **(B)** drug-gene interaction predicted *via* DGIdb database.

In the GSE66239 dataset, a total of 471 DEmiRNAs were identified, including 194 upregulated and 277 downregulated miRNAs (**Supplementary Figure S5A**). Considering that all five hub genes were upregulated, we only extracted downregulated DEmiRNAs for subsequent analysis. The flowchart of ceRNA network construction is displayed in **Supplementary Figure S5B**. According to the integrated lncRNA-miRNA-mRNA analysis pipeline we proposed above, five ceRNA networks were constructed to decipher the potential post-transcriptional regulation mechanism of hub genes (**Figure 4A**). In each network, lncRNAs may promote the expression of hub genes by inhibiting the corresponding miRNAs. For instance, *CDKN2B-AS1* may inhibit has-*miR-6827-5p* and thus upregulate the expression of *IL6*. Notably, *LINC00960* might be able to affect both *CXCL10* and *STAT1* through multiple miRNAs, including a consensus *has-miR6801-5p*. Likewise, *IL10* and *VEGFA* shared four lncRNA regulators encompassing *LINC1001, CTD-2006K23.1, RP4-647J21.1*, and *CSNK1G2-AS1*, which may be vital RNA agents or targets candidate for anti-inflammatory and proendothelialization molecular therapy. In addition, the latent drugs interacting with hub genes were also explored in the DGIdb database. The *IL6* inhibitors Olokizumab and Siltuximab, and the *VEGFA* inhibitors Aflibercept, Bevacizumab, Ranibizumab, and Pegaptanib Sodium were predicted to serve as potential therapeutic agents for IAs (**Figure 4B**).

### Gene Co-Expression Modules With Regard to Immune Infiltration

**Figure 5:**
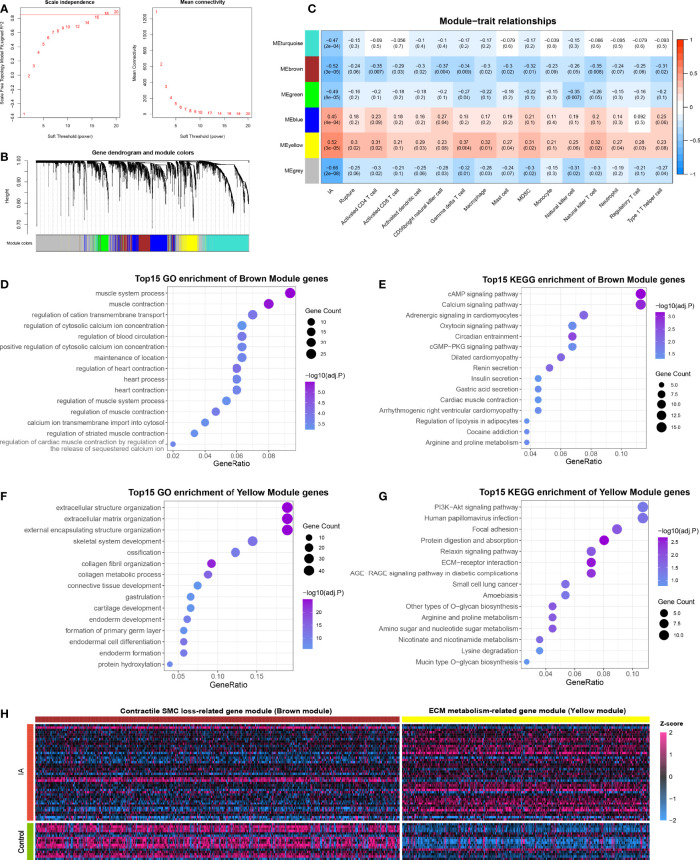
Functional gene co-expression modules are recognized by WGCNA. **(A)** Determination of the soft-thresholding. **(B)** Gene clustering and co-expression module recognition. **(C)** Heatmap of the association of gene modules with IA, rupture status, and immune cell infiltration. **(D, E).** GO **(D)** and KEGG **(E)** enrichment analysis of brown module genes. **(F, G).** GO **(F)** and KEGG **(G)** enrichment analysis of yellow module genes. **(H)** Heatmap of DEGs in brown and yellow modules.

The 3310 DEGs between IA and control were included in the WGCNA analysis, and the soft power of β =18 (scale-free R^2 =^ 0.89) was determined as soft-thresholding to acquire DEG co-expressed gene modules (**Figure 5A**). Ultimately, 739,394,179,509 and 268 DEGs were clustered into turquoise, brown, green, blue, and yellow modules, respectively; while the other 1221 genes were assigned to the grey region due to failed clustering. (**Figure 5B**). Among these gene modules, yellow module was significantly positively associated with aneurysm rupture (*r* =0.3, *P* =0.02) and nine vital immune cells, such as γδ T cell (*r* =0.37, *P* =0.004) and macrophage (*r* =0.32, *P* =0.01, **Figure 5C**). Brown module was remarkably negatively correlated with ten vital immune cells such as CD56+ NK cell (*r* = -0.37, *P* =0.004) and macrophage (*r* = -0.3, *P* =0.02), and this module also presented a potential negative correlation with rupture status, although the *P*-value is not perfectly significant (*r* = -0.24, *P* =0.06). Taken together, these two modules were positively or negatively relevant to the most immune cells and were considered important transcriptionally dysregulated gene modules with regard to the altered immune microenvironment.

To further ascertain the biological function of the modules, GO and KEGG enrichment analyses were conducted for genes in the brown and yellow modules, respectively. Brown module genes were pronounced enriched in muscle contraction, regulation of cytosolic calcium ion concentration, calcium ion transmembrane import into the cytosol, cAMP signaling pathway, calcium signaling pathway, and so on, which can presumably be summarized as SMC contraction-related gene module (**Figures 5D, E**). Likewise, the yellow module was characterized as ECM metabolism-related gene modules, which were mainly enriched in the extracellular matrix organization, external encapsulating structure organization, collagen metabolic process, PI3K-Akt signaling pathway, ECM-receptor interaction, etc. (**Figures 5F, G**). Considering that yellow and brown modules contained mainly up- and down-regulated DEGs, respectively, we speculated that the altered immune microenvironment may accompany and even mediate two other essential pathologies in IA: contractile SMC dysfunction or loss, and ECM degradation and remodeling (**Figure 5H**).

### PFDGS Generated From Combined Machine Learning Algorithms

**Figure 6:**
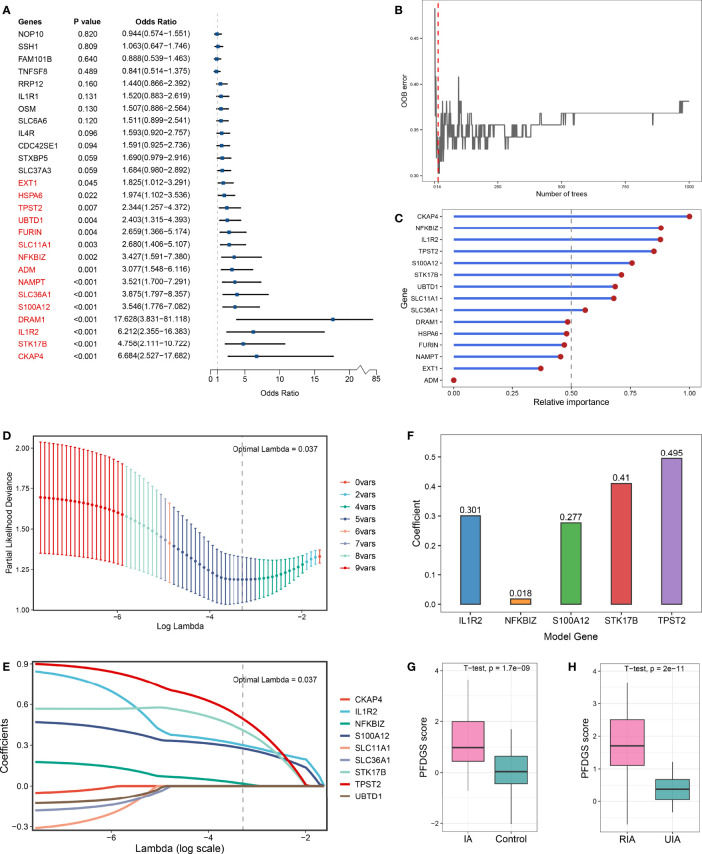
Integrative construction of the PFDGS in blood cohort. **(A)** Univariate Logistic regression analysis of the 27 shared DEGs in blood and tissue. **(B)** Out of bag (OOB) error rate reached a minimum when the number of trees was equal to 14. **(C)** Relative importance of 15 initial candidate marker genes calculated in random forest. **(D)** The optimal lambda was determined when the partial likelihood deviance reached the minimum value. **(E)** LASSO coefficient profiles of the candidate genes for PFDGS construction. **(F)** Coefficients of 5 model genes in PFDGS. **(G, H)** Comparison of the PFDGS scores between IAs and controls **(G)** and between RIAs and UIAs **(H)**.

Taken together, upregulated immune-related DEGs, contractile SMC loss-related DEGs, and ECM metabolism-related DEGs were considered essential pathological feature-related genes in IA, which may serve as promising biomarkers. To explore the diagnostic and predictive value of these genes and enhance their clinical applicability, we attempted to pursue shared DEGs in peripheral blood as candidate biomarkers. As a result, 27 DEGs with consistent alteration trends in blood and tissue were detected in the meta-cohort. Subsequently, the expression profiles of these 27 DEGs were subjected to the machine learning-based procedure to develop a pathological feature-derived gene signature (PFDGS). Univariate Logistic regression analysis of the 27 DEGs revealed 15 marker genes that were also significantly associated with IA rupture (**Figure 6A**), followed by random forest, screening out nine genes with relative importance greater than 0.5 (**Figures 6B, C**). The nine genes were incorporated into LASSO regression to generate the final PFDGS model. Based on the 5-fold cross-validation, the model reached an optimum when lambda was equal to 0.037, containing five key gene variables (**Figures 6D, E**). A risk score for each patient was calculated based on the expression of the five model genes with detailed formula as follow: PFDGS = 0.301*IL1R2 + 0.018*NFKBIZ + 0.277*S100A12 + 0.410*STK17B + 0.495*TPST2 + 0.772 (**Figure 6F**).

### Diagnostic and Predictive Value of the PFDGS Model

**Figure 7:**
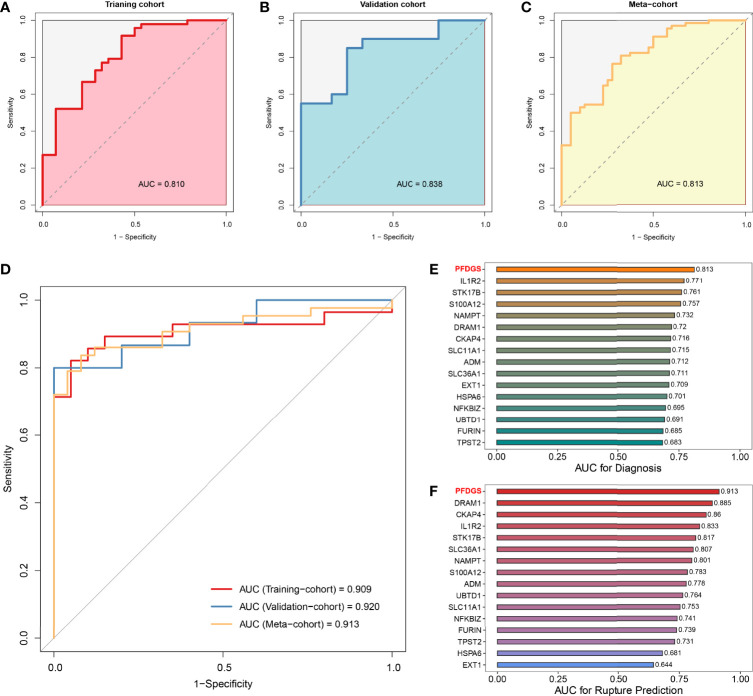
Diagnostic and predictive value of PFDGS. **(A-C).** ROC analysis of the PFDGS for IA diagnosis in the training cohort **(A)**, validation cohort **(B)**, and meta-cohort **(C)**. **(D)** ROC analysis of the PFDGS for predicting IA rupture in the training cohort, validation, and meta cohorts. **(E, F).** AUC values of PFDGS and 15 initial candidate marker genes for IA diagnosis **(E)** and rupture prediction **(F)** in the meta-cohort.

Patients with IA presented higher PFDGS scores than IA-free controls (**Figure 6G**); and when compared between RIAs and UIAs, PFDGS was relatively higher in RIAs (**Figure 6H**). To quantitatively assess the diagnostic and predictive value of the model, we conducted an ROC curve analysis of PFDGS and 15 initial candidate marker genes. When IA patients were set as positive and the controls as negative, the AUC of PFDGS in the training cohort (n =76), validation cohort (n =32), and meta-cohort (n =108) were 0.810, 0.838, and 0.813, respectively, indicating that the PFDGS had a good performance in the diagnosis of IA (**Figures 7A-C**). Likewise, when patients with RIA were set as positive and patients with UIA as negative, the AUC of PFDGS in the training cohort (n =48), validation cohort (n =20), and meta-cohort (n =68) were 0.909, 0.920, and 0.913, respectively, suggesting that the PFDGS also possessed a great performance in predicting IA rupture (**Figure 7D**). Moreover, PFDGS also displayed superior AUC values relative to 15 initial candidate marker genes alone for diagnosis or prediction, which implied the superiority of the gene-combined signature (**Figures 7E, F**). Overall, patients with high PFDGS scores may possess a higher risk of IA onset or rupture, requiring more vigilant attention or timely interventional treatment.

### Biological Implications Underlying PFDGS

**Figure 8:**
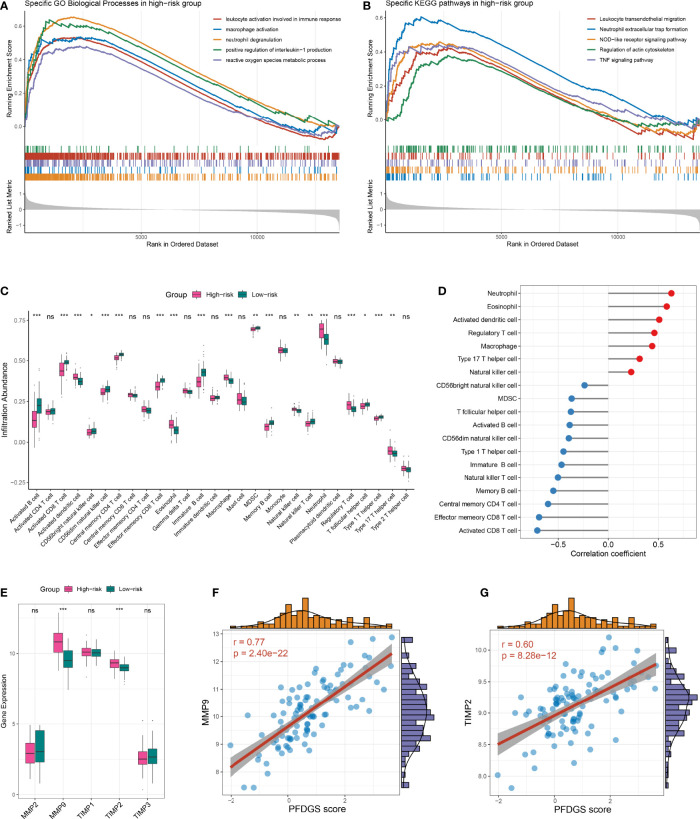
Biological significance underlying the PFDGS. **(A, B).** GO **(A)** and KEGG **(B)** gene set enrichment analysis were performed between high- and low-risk groups. **(C)** Comparison of immune cell relative abundance between high- and low-risk groups. **(D)** Relationship between PFDGS and immune cell abundance. **(E)** Comparison of MMP2, MMP9, TIMP1, TIMP2, and TIMP3 expression between high- and low-risk groups. **(F, G).** Correlation of PFDGS with MMP9 **(F)** and TIMP2 **(G)** expression. For all boxplots, ns, *P >*0.05; *P < 0.05, **P < 0.01, ***P < 0.001.

To further investigate the potential biological mechanisms underlying the PFDGS, we divided all samples in the meta-cohort into high- and low-risk groups according to the median PFDGS score and performed GSEA analysis. The high-risk group enriched numerous pathways associated with immune-inflammatory activation and metabolic disorders, such as leukocyte activation involved in immune response, macrophage activation, neutrophil extracellular trap formation, positive regulation of interleukin-1 production, NOD-like receptor signaling pathway, TNF signaling pathway, reactive oxygen species metabolic process, and regulation of actin cytoskeleton (**Figures 8A, B**). We subsequently measured immune cell abundance based on blood gene expression profile using ssGSEA, and detected 19 differentially infiltrated immune cells between high- and low- risk groups (**Figure 8C**). Of note, the high-risk group presented more abundant myeloid cells (e.g., neutrophils, eosinophils, and macrophages) but few lymphocytes (e.g., activated CD8 T cells, immature B cell, and NK T cell) relative to the low-risk group. This is similar to the special phenomenon of increased inflammatory cells but decreased lymphocytes in the peripheral blood of IA patients discovered in recent years. PFDGS was positively or negatively correlated with up- and down-regulated immune cells, respectively (**Figure 8D**), implying an ability of PFDGS to reflect the special alteration of immune cells in the peripheral blood of IA patients. In addition, we also investigated the association of PFDGS with matrix metalloproteinases (MMPs) and tissue inhibitors of metalloproteinases (TIMPs), two essential molecular clusters involved in ECM metabolism (34). In the meta-cohort, five genes encoding MMPs and TIMPs were detected, and *MMP9* and *TIMP2* were significantly upregulated in the high-risk group (**Figure 8E**). Correlation analysis indicated that PFDGS was positively associated with the expression of *MMP9* (*r* =0.77, *P <*0.001) and *TIMP2* (*r* =0.60, *P <*0.001; **Figures 8F, G**), suggesting the potential of PFDGS to reflect ECM metabolism. Taken together, a high PFDGS score represented multiple adverse biological alterations, including a high level of immunoinflammatory activation, disordered peripheral blood myeloid cells and lymphocytes, and a high level of ECM hypermetabolism, which may contribute to the biological interpretation that patients with high PFDGS scores possessed a higher risk of IA onset and rupture.

## Discussion

IA is an uncommon but life-threatening cerebrovascular disease with incompletely ascertained pathological mechanisms. Immunoinflammatory is believed to be an essential lesion contributor in the progress of IA formation and rupture as well as the presence of complications after endovascular treatment (35, 36). However, the limited knowledge of cellular or molecular alterations in the immune microenvironment of IA hampered the diagnostic and therapeutic technique improvement, especially drug-coated stent-based device amelioration and molecular test-based individualized assessment. In this study, we systematically unveiled an “mmune-thermal” microenvironment encompassing aberrant immune cells, and immune-related pathways and molecules, which may be vital immune activation mediators and promising therapeutic targets in IA. More specifically, our study identified five immune-related hub genes and revealed corresponding ceRNA regulatory networks and latent drugs, providing a novel reference for mechanism understanding and targeted therapy of IA. Using the advanced WGCNA algorithm, two essential gene co-expression modules with respect to altered immune infiltration were recognized and functionally annotated as contractile SMC loss-related and ECM metabolism-related modules. According to the DEGs associated with the abovementioned pathological features, our study preliminarily established a blood-based PFDGS model for detecting IA patients at high risk of morbidity or aneurysm rupture, which may serve as an attractive biomarker in clinical management.

Aberrant immune microenvironment plays essential roles in the formation, progression, and rupture of IAs. Using several bioinformatics algorithms, our study revealed multi-level alterations of the IA microenvironment, comprising abundant immune cell infiltration, activated immune-related pathways, and high expression of immune-related genes, which can be summarized as “immuno-thermal”. Notably, concomitant with immune activation, immunosuppressive cells, such as MDSC and Treg, and anti-inflammatory cytokines, such as *IL10*, are also significantly highly infiltrated or upregulated in IA. This phenomenon is similar to the concept of “concurrent hyperinflammation and immune suppression” proposed by van der Poll et al. in sepsis (37). Both immune activation and suppression are enhanced but unbalanced, globally manifesting as pathogenic inflammation. Recently, a single-cell atlas of human abdominal aortic aneurysm and carotid atherosclerosis was revealed, which also detected the phenomenon of pro-inflammatory and anti-inflammatory cell co-proliferation (38, 39). Taken together, we reasoned that the “immuno-thermal” microenvironment of IA was characterized by co-enhanced immune activation and immunosuppression, constituting a complex pathological feature.

More concretely, our study identified 14 abnormally high infiltrated immune cells in IA tissues. Among these cells, macrophages have attracted much attention and are considered to be crucial lesion driver immune cells in IAs, due to their roles in immune recruitment, inflammatory injury, and ECM degradation promotion (40, 41). Moreover, macrophages also serve as favored therapeutic target cells because of their ability to modulate immunity *via* pro-inflammatory (M1) and anti-inflammatory (M2) phenotypic polarization. Another function of macrophages that has received little attention in IA is the detection of molecular signals of cell damage and death, known as damage-associated molecular patterns (DAMPs) (42). Antigen presentation cells such as macrophages and DCs detected DAMP through toll-like receptors (TLRs) and then presented DAMP on major histocompatibility complex II (MHC II), which can further mediate lymphocyte maturation and activation (43). In this study, we detected that activated CD4 T cell, activated CD8 T cell, γδ T cell, Th1 cell, and Treg cell were positively correlated with several innate immune cells, implying a possible link or even crosstalk between innate and adaptive immune cells existed in IA. Consistent with our findings, Juhana et al. have performed a histological analysis and evidenced that great numbers of macrophages and T lymphocytes infiltrated in IA and were associated with aneurysm rupture (44). Recently, the potential roles of mast cells and neutrophils in IA have also been reported. Mast cells related to intramural microhemorrhage and wall degeneration, as well as neutrophil extracellular traps (NETs) involved in inflammation and tissue remodeling, both contribute to the increased risk of aneurysm rupture (45, 46). For NK cells and MDSC, the perception of their roles in IA pathological processes is extremely limited. Studies on similar diseases have reported that increased NK cells and cytotoxicity are associated with causing or exacerbating abdominal aortic aneurysm inflammation (47), and MDSCs are able to reduce atherosclerosis *via* inhibiting pro-inflammatory responses (48). Nevertheless, the role of NK cells and MDSC in IA still needs to be determined in future experiments.

With the continuous progress of drug-delivery technology, biologics-based immunotherapy is exerting an essential role against major inflammatory diseases, such as cancer and vascular inflammation (49). Particularly, endovascular devices carrying therapeutic agents *via* drug coating or elution techniques are considered promising modifications in IA treatment (50). For instance, CD31-mimetic-coated flow-diverters can promote IA healing and improve stent biocompatibility *via* accelerating endothelialization and reducing leukocyte accumulation (17). In our study, a series of dysregulated immune cells, immune-related signaling pathways, as well as five immune-related hub genes were identified, providing a potential target reference for therapeutic immune modulation. Among the five hub genes, *CXCL10, IL6, STAT1*, and *VEGFA* are well-known factors involved in immune recruitment or activation, while *IL10* generally acts as an inhibitory immunomodulator. Hence, the predicted ceRNA molecules and latent drugs, containing the miRNAs targeting *CXCL10, IL6, STAT1*, and *VEGFA*, the lncRNAs indirectly regulating *IL10*, the *IL6* inhibitors Olokizumab and Siltuximab, as well as the *VEGFA* inhibitors Aflibercept, Bevacizumab, Ranibizumab, and Pegaptanib Sodium, may represent valuable immunotherapeutic agents for ameliorating excessive inflammation in IA. Of note, *VEGFA*-targeted therapies may display a dual role. Some studies suggest that aneurysmal disease requires anti-*VEGFA* therapy to reduce inflammation and tissue destruction (51), whereas recent studies suggest that promoting *VEGFA* expression may contributed to accelerating endothelialization after stent implantation (52, 53). Therefore, the above immunomodulators still need more functional experimental exploration and rigorous trade-offs prior to therapeutic application.

Current detection and risk assessment of IA primarily rely on imaging and morphological characteristics, and lack assistance with molecular pathological markers. In the present study, we systematically identified a blood-based gene risk signature derived from DEGs associated with three aspects of IA pathological features, encompassing immune activation, contractile SMC loss, as well as ECM hypermetabolism. The diagnostic and predictive value analysis indicated that our PFDGS model possessed high accuracy and stable performance for both IA diagnosis and rupture prediction in the training cohort, validation cohort, and meta-cohort, which implied a great potential of the PFDGS for clinical translation. Of note, PFDGS displayed higher accuracy compared with the 15 candidate biomarker genes irrespective of its use for diagnosis or prediction, which was consistent with the multimolecular driving nature of IA. Furthermore, a high PFDGS score represented a higher level of immunoinflammatory activation, peripheral blood myeloid cells and lymphocytes disturbance, and ECM hypermetabolism, which suggested that the PFDGS can also serve as an indicator of deleterious molecular pathological alterations in IA patients. Overall, subjects with high PFDGS scores may possess adverse biological alterations and present with a higher risk of IA morbidity or aneurysm rupture, requiring more vigilant attention or timely therapeutic intervention.

Although the present study systematically revealed the immune microenvironment of IA and determined a promising gene signature for IA risk estimation, certain limitations should also be acknowledged. First, the decoding of immune cells in this study was based on total RNA sequencing data of IA tissues, and future sequencing at the single-cell level may provide a more accurate cell microanatomy of IA. Second, the PFDGS has only been validated in small samples of public data, and prospective clinical verification with larger samples is required prior to clinical application. Third, the regulatory mechanism of ceRNA networks needs further functional experiments.

In conclusion, the present study systematically unveiled an “immuno-thermal” microenvironment characterized by co-enhanced immune activation and immunosuppression in IA, which provided a novel insight into IA molecular pathology. Aberrant immune cells and genes were shown to be vital target candidates for molecular therapy. The PFDGS serves as a promising signature for the detection of patients at high risk of IA morbidity and aneurysm rupture, which can facilitate risk surveillance and clinical decision-making in IA patients.

## Data Availability Statement

The datasets presented in this study can be found in online repositories. The names of the repository/repositories and accession number(s) can be found in the article/**Supplementary Material**.

## Ethics Statement

The studies involving human participants were reviewed and approved by the Ethical Committee of Zhengzhou University People’s Hospital. The patients/participants provided their written informed consent to participate in this study.

## Author Contributions

TYL and YKH designed this work. TYL and ZQL integrated and analyzed the data. DHG, YYH, TYL, CM, YYL and RFJ performed the experiments. TYL wrote this manuscript. TXL, YKH, ZQL, TYL, DHG, YYH, LD, CGG, ZX, and YYL edited and revised the manuscript. All authors contributed to the article and approved the submitted version.

## Funding

This study was supported by the Young and Middle-aged Health Science and Technology Innovation Talent Training Project of Henan Province (Grant No. YXKC2020041); and the National Key Research and Development Program of the 13th Five-Year Plan of China (Grant No. 2016YFC1300702).

## Conflict of Interest

The authors declare that the research was conducted in the absence of any commercial or financial relationships that could be construed as a potential conflict of interest.

## Publisher’s Note

All claims expressed in this article are solely those of the authors and do not necessarily represent those of their affiliated organizations, or those of the publisher, the editors and the reviewers. Any product that may be evaluated in this article, or claim that may be made by its manufacturer, is not guaranteed or endorsed by the publisher.
