# Supplementary material for: Transcriptome-Based Dissection of Intracranial Aneurysms Unveils an “Immuno-Thermal” Microenvironment and Defines a Pathological Feature-Derived Gene Signature for Risk Estimation
Source: Front Immunol. 2022 May 31;13:878195. doi: 10.3389/fimmu.2022.878195 (PMC9194475; doi:10.3389/fimmu.2022.878195)
Supplement: Supplementary file 1 [file DataSheet_1.docx]

**Supplementary Figures**

**Figure S1.** Before and after removing batch effects from non-biological technical biases.

**Figure S2.** Analysis of immune cell infiltration in different datasets.

**Figure S3.** Correlation heatmap of immune cell infiltration abundance.

**Figure S4.** GO and KEGG enrichment analysis of immune-related DEGs.

**Figure S5.** Construction process of the ceRNA networks.

**
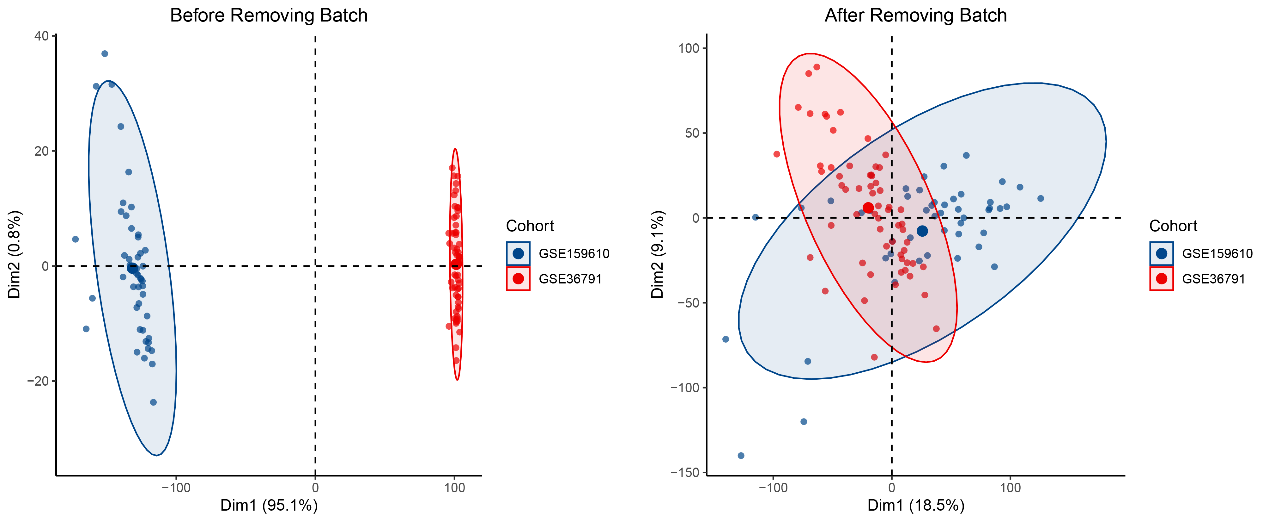
**

**Figure S1.** Before and after removing batch effects from non-biological technical biases.

**
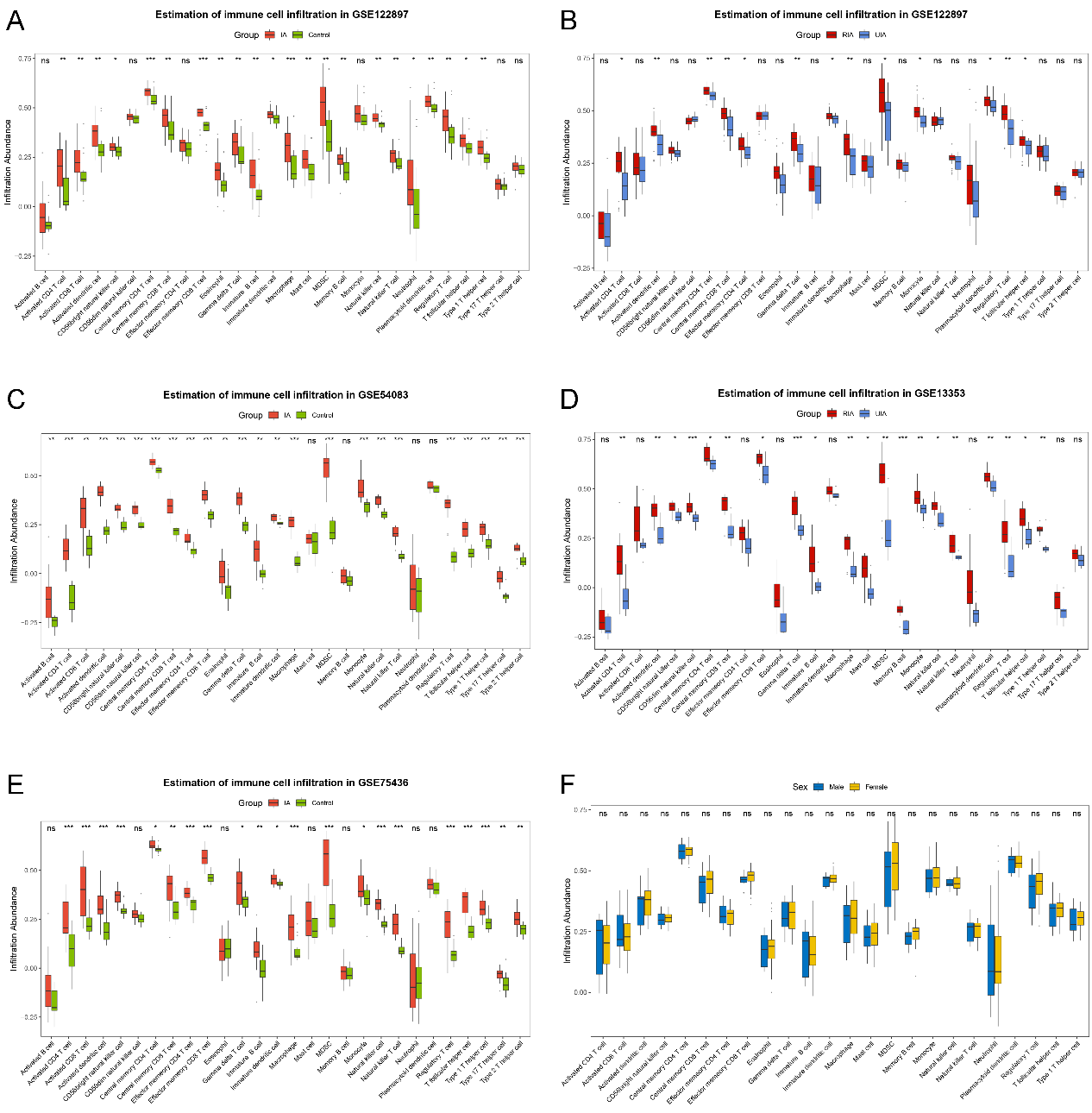
**

**Figure S2.** Analysis of immune cell infiltration in different datasets. **A-B.** Comparison of immune cell infiltration between IAs and controls (**A**) and between RIAs and UIAs (**B**) in GSE122897. **C.** Comparison of immune cell infiltration between IAs and controls in GSE54083. **D.** Comparison of immune cell infiltration between RIAs and UIAs in GSE13353. **E.** Comparison of immune cell infiltration between IAs and controls in GSE75436. **F.** Comparison of immune cell infiltration between male and female in GSE122897. For all boxplots, ns, *P* >0.05; **P* < 0.05, ***P* < 0.01, ****P* < 0.001.)


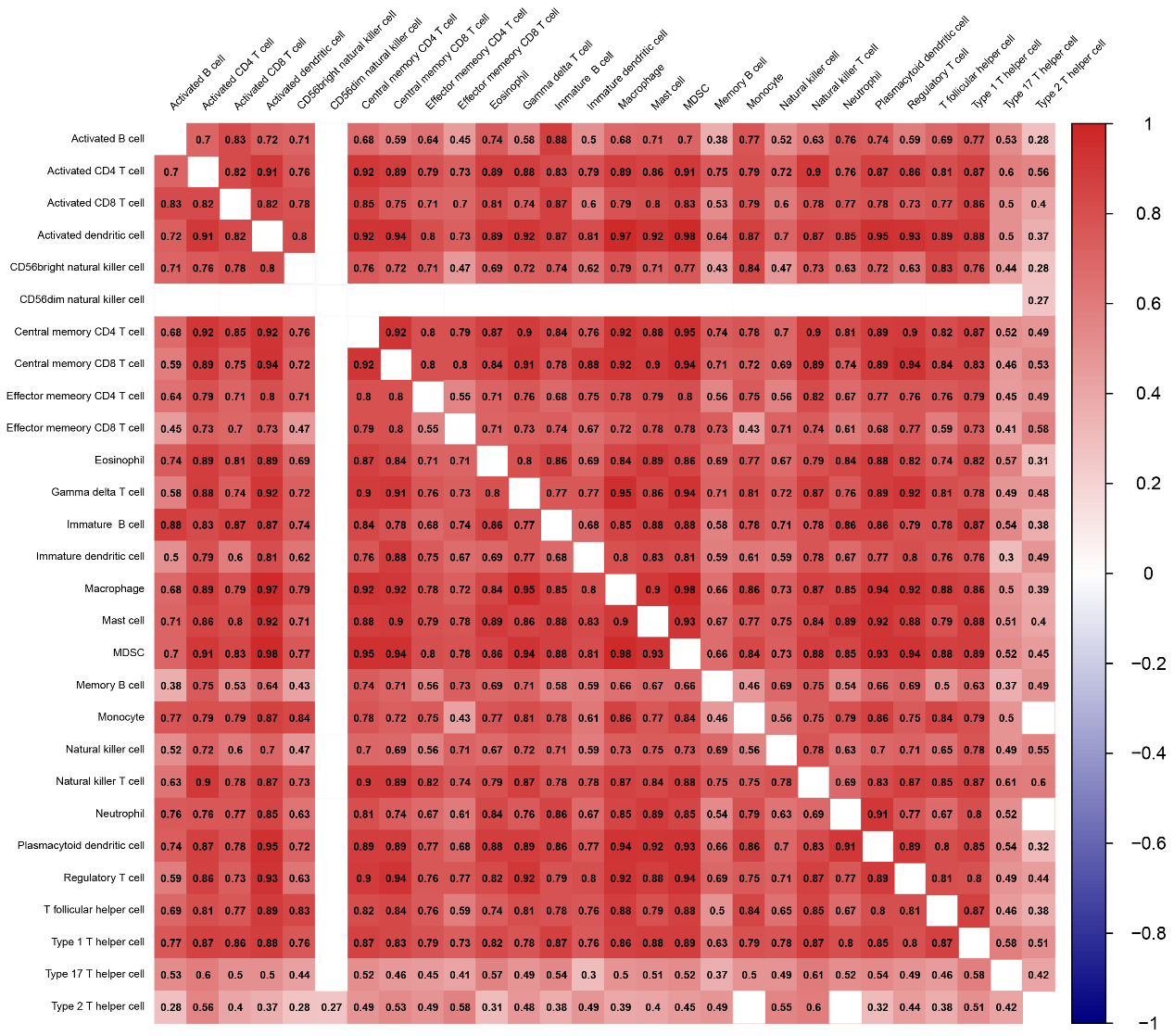


**Figure S3.** Correlation heatmap of immune cell infiltration abundance. Red represents positive correlation, blue represents negative correlation, and white represents insignificant (P > 0.05) or autocorrelation (correlation coefficient = 1)

**
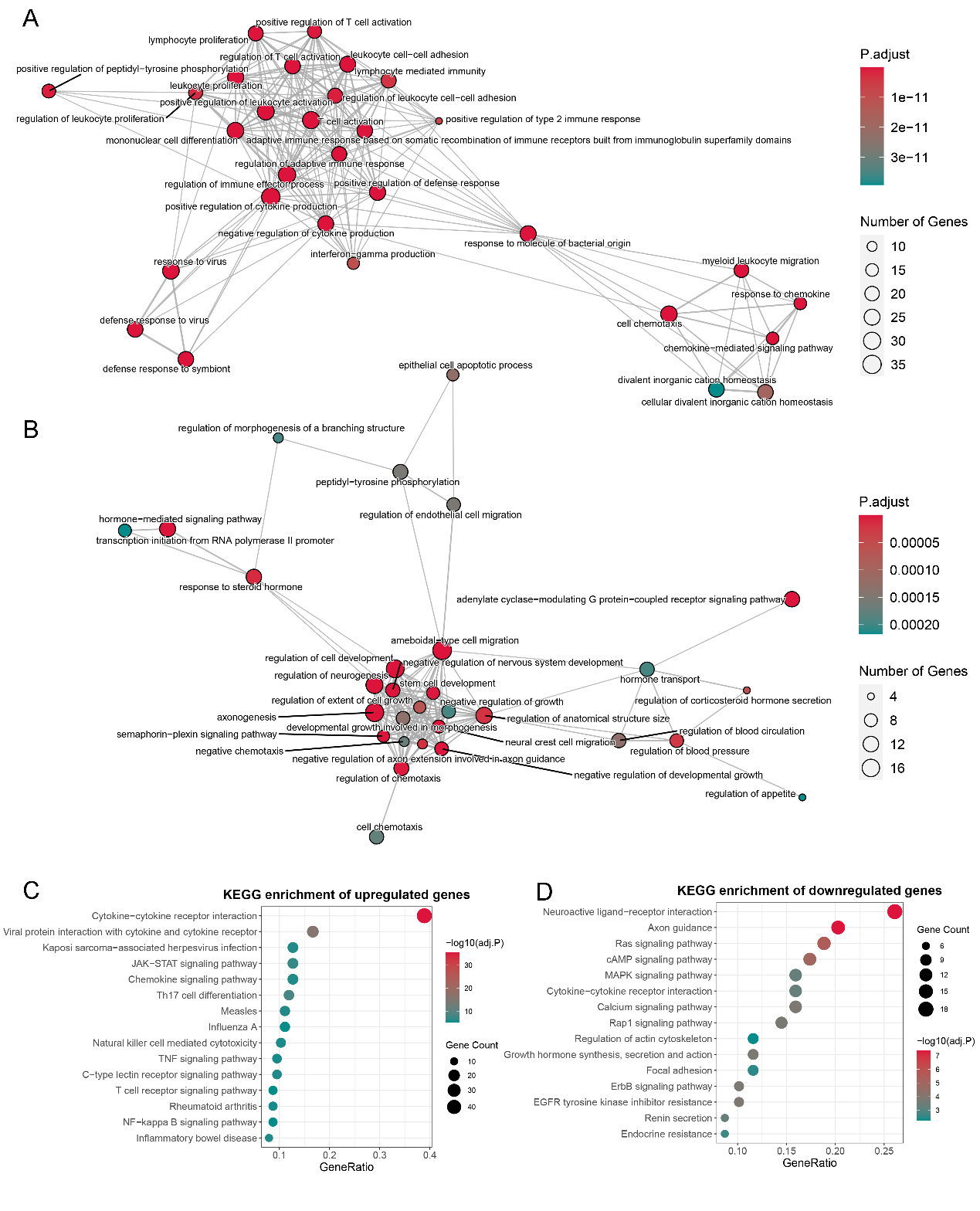
**

**Figure S4.** GO and KEGG enrichment analysis of immune-related DEGs. **A-B.** GO enrichment analysis of upregulated (A) and downregulated (B) immune-related genes. **C-D.** KEGG enrichment analysis of upregulated (C) and downregulated (D) immune-related genes.

**
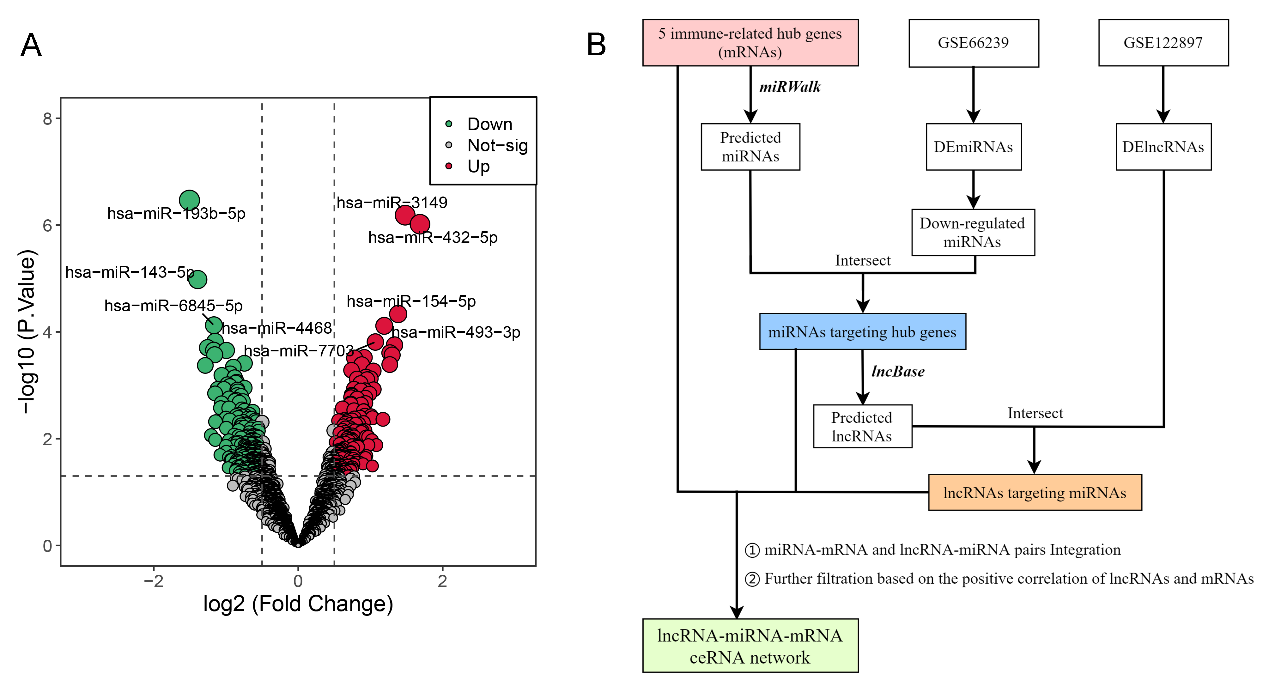
**

**Figure S5.** Construction process of the ceRNA networks. **A.** Volcano plot of DEmiRNAs between IA and control. Red dots represent significantly up-regulated DEGs and green dots represent significantly down-regulated DEGs. **B.** Flow chart for the construction of ceRNA network.
